# A novel classification based on B-cell receptor signal gene expression correlates with prognosis in primary breast diffuse large B-cell lymphoma

**DOI:** 10.7150/jca.39083

**Published:** 2020-02-10

**Authors:** Wenjia Su, Xingjian Niu, Hongfei Ji, Yang Xu, Lei Zhong, Shuye Wang, Dabei Tang, Xiaoping Zhou, Qingyuan Zhang, Jin Zhou

**Affiliations:** 1 Department of Hematology, First Affiliated Hospital of Harbin Medical University, Harbin Medical University, Harbin 150081, Heilongjiang, China; 2 Department of Medical Oncology, Harbin Medical University Cancer Hospital, Harbin Medical University, Harbin 150081, Heilongjiang, China; 3 Institute of Cancer Prevention and Treatment, Harbin Medical University, Harbin 150081, Heilongjiang, China; 4 Heilongjiang Academy of Medical Sciences, Harbin 150081, Heilongjiang, China; 5 Department of General Surgery, Second Affiliated Hospital of Harbin Medical University, Harbin Medical University, Harbin 150081, Heilongjiang, China

**Keywords:** Primary breast diffuse large B-cell lymphoma, B-cell receptor signaling pathway, Cluster analysis, Prognosis

## Abstract

Primary breast diffuse large B-cell lymphoma (PB-DLBCL), the most common histologic subtype of lymphoid malignancy in the breast, is a clinically and genetically heterogeneous disease that has insufficient systematic studies on the pathological and molecular features, optimal treatment scheme, as well as the prognostic factors. The aim of our study was to identify biomarkers and distinct subtypes of PB-DLBCLs and then evaluate the prognosis of this rare malignant lymphoma. We carried out hierarchical clustering analysis to evaluate protein expressions of potential biomarkers detected by immunohistochemistry staining of samples from 68 PB-DLBCL patients. The gene expression data from TCGA database was obtained to validate the identified clusters. We identified three robust clusters based on the B-cell receptor (BCR) signaling pathway, including two recognized NF-κB-dependent and PI3K-dependent clusters, and a distinct subset of PB-DLBCL with NF-κB-independent anti-apoptotic overexpression plus PI3K signaling, which exhibited an evolving definition and distinctive characters of a cluster group. Furthermore, survival analysis results showed an inferior outcome in NF-κB-dependent cluster patients and favorable survival in the PI3K-dependent cluster patients, suggesting an important predictive value of the three clusters. Our study provided a new perspective for understanding clinical complexity of PB-DLBCLs, and gave evidence for finding targeted biomarkers and strategies.

## Introduction

Diffuse large B-cell lymphoma (DLBCL) is the most common aggressive lymphoma subtype with distinct genetic backgrounds and clinical characteristics 1-3. Due to the biological heterogeneity of DLBCL, the responses to therapy and the prognostic survivals of patients are also different 1, 3, 4. Currently, the cell-of-origin (COO) classification [activated B-cell like (ABC) and germinal center B-cell like (GCB)] and the International Prognostic Index (IPI) score are two most commonly used prognostic factors for DLBCL patients 4-6. However, these predictors do not completely elucidate the risk stratification, variable outcomes, as well as the complex mechanisms underlying tumor biology of DLBCL 7. The latest findings based on whole-exome sequencing have identified five robust DLBCL subsets which linked genetic signatures with pathogenetic mechanisms 8. More importantly, these newly defined DLBCL subsets provided new insights into assessment of clinical outcomes and more rational therapies 8.

Up to a third of DLBCLs arise from extranodal organs, which are usually characterized by poor survival 9. DLBCL primary of the breast (PB-DLBCL) are a rare presentation of extranodal DLBCL, representing less than 2.0% of all cases 10-12. Because of the rare incidence of PB-DLBCL, there exists limited data on the natural history of this lymphoma entity 13-15. Generally, PB-DLBCL mainly shows a phenotype of ABC (60-90%) according to the COO classification 13, 16. However, as an uncommon site of involvement in extranodal DLBCL, PB-DLBCL has its own particularity with heterogenic biological and clinical characters 16. In addition, PB-DLBCL has been reported to exhibit a worse prognosis compared with other extranodal DLBCLs, and the 5-year overall survival (OS) rates are nearly 50% 17. Therefore, valuable biomarkers based on profound understanding the distinct subtypes are warranted to guide prognostic factors and therapeutic approaches for PB-DLBCL.

The genetic heterogeneity is usually reflected by gene-expression profiling, in which B cell receptor (BCR) signaling pathway plays the key role in DLBCL 18-20. BCR signaling pathway mediates the survival signals in almost all DLBCL cells, including “chronic active” and “tonic” BCR signaling 18. Previous studies have shown that extranodal DLBCL cells were mainly derived by chronic active BCR signaling with selectively acquiring mutations that target the BCR 21. Active BCR signaling engages many complex transcriptional networks and pathways. After ligand binding, BCRs cluster, BCR pathway adaptor caspase recruitment domain family member 11 (CARD11), and resultant protein tyrosine kinases (PTK) will recruit and activate, thus initiate downstream NF-κB signaling pathway mainly 19, 22, 23. BCR-pathway components and networks are complex and variable, which may provide the basis of underlying the biological diversity of the PB-DLBCL. More importantly, comprehensive establishment of the expression profile of PB-DLBCL will be helpful for classifying the distinct subsets and determining the subtype-specific signaling targets, treatments as well as the outcomes of PB-DLBCL. Herein, we identified potential subtypes by performing clustering analysis of the downstream components of BCR signaling pathways in PB-DLBCL patients, and then characterized each cluster in order to predict therapeutic effects as well as the prognostic survival of PB-DLBCL patients.

## Methods

### Patients

We retrospectively studied 68 cases of female patients with PB-DLBCLs, of whom 50 cases were treated in Harbin Medical University Cancer Hospital, 6 cases in the First Affiliated Hospital of Harbin Medical University and 12 cases in the Second Affiliated Hospital of Harbin Medical University from June 1976 through December 2016. Patients who fulfilled the following criteria were included in our current study: (1) diagnosed with primary breast lymphomas (PBLs) according to the criteria of Wiseman and Liao 24: adequate pathologic specimen technically and close association of lymphomatous infiltration and breast tissue; (2) histologic classification of DLBCL according to the 2016 World Health Organization (WHO) criteria 25. Patients were excluded if they were in the following cases: (1) a previous history of extramammary lymphoma or indolent B-cell lymphoma; (2) post-transplant lymphoproliferative disorders; (3) receiving major surgery within 4 weeks; (4) uncontrolled systemic infection; (5) EBV-positive DLBCL; (6) serological positivity for Hepatitis B, C virus or HIV infection. Ethical protocol was approved by the Institutional Review Board of Harbin Medical University and written informed consents were obtained from the patients or guardians. All methods were performed in accordance with the relevant guidelines and regulations. The pretreatment workups included a complete history and physical examination. We collected the histological types and various clinical or laboratory parameters.

### Immunohistochemistry (IHC) staining and data analysis

Formalin-fixed, paraffin-embedded tissue specimens were collected from 68 patients diagnosed with PB-DLBCL patients for IHC analysis. Briefly, tissue sections were de-paraffinized in xylene and rehydrated in grade ethanol. High-pressure antigen retrieval was conducted using citrate buffer (pH 6.0). Next, endogenous peroxidase activity was blocked using 3% H_2_O_2_. Subsequently, the slides were incubated with single primary antibody respectively [anti-CD10 antibody (dilution 1:200); anti-BCL6 antibody (dilution 1:200); anti-MUM1 antibody (dilution 1:200); anti-PI3K antibody (dilution 1:300); anti-AKT2 antibody (dilution 1:200); anti-JAK2 antibody (dilution 1:300); anti-STAT3 antibody (dilution 1:300); anti-MAPK antibody (dilution 1:300); anti-BCL10 antibody (dilution 1:500); anti-NF-κB (p50) antibody (dilution 1:200); anti-Myc antibody (dilution 1:200); anti-BCL2 antibody (dilution 1:300); anti-MCL1 antibody (dilution 1:200); anti-BCL-xL antibody (dilution 1:200); anti-Ki67 antibody (dilution 1:300); and anti-P53 antibody (dilution 1:100), Abcam, Cambridge, USA)] at 4°C overnight. Then the slides were further incubated with HRP-labelled secondary antibody (Abcam, Cambridge, USA) for 30 min at room temperature. Then the slides were counterstained with instant hematoxylin, and then dehydrated, cleared, and mounted. All tissue specimens were examined by three independent well-experienced pathologists in a blinded manner (200 × magnification) without any prior information of the patient samples. According to a commonly used standard for IHC staining in DLBCL defined by Hans et al 26, the highest percentage of stained tumor cells was calculated to decide the positive cells in each case, and positive expression result was based on the cut-off value. The cut-off values for the proteins were shown as reported previously 7, 26-32: CD10 (30%), BCL6 (30%), MUM1 (30%), PI3K (30%), AKT2 (20%), JAK2 (30%), STAT3 (30%), MAPK (20%), BCL10 (20%), NF-κB p50 (nuclear 20%), Myc (40%), BCL2 (50%), MCL1 (50%), BCL-xL (50%), Ki67 (70%) and P53 (30%) respectively. Staining intensity of each tissue sections were also evaluated, but it was not applied for determining the positivity due to the variability in tissue fixation and processing according to the previous study 26. Phosphate buffered saline was used as negative control. The expression data of these proteins were evaluated by hierarchical clustering analysis using Manhattan Distance Method in Multi Experiment View (MEV) cluster software.

### Gene expression data from TCGA database

The gene expression data and detailed clinical information from TCGA database (https://cancergenome.nih.gov/) were obtained and enrolled. The values of gene expressions from the DLBCL data sets were standardized by log 2. Hierarchical clustering analysis using Euclidean Distance Method was performed to assess TCGA data by Multi Experiment View (MEV) cluster software.

### Statistical analysis

The last follow-up was in June 2017. The Kaplan-Meier methods was used to evaluate the OS, defined as the period of time from the date of diagnosis to the date of death from any causes or last follow-up. Different groups were compared using the log-rank test. Chi-squared and Fisher's Exact tests were carried out to evaluate the associations between the clusters and clinicopathological parameters. Multivariate analysis to evaluate the variables was performed using Cox proportional hazards models, and the results were presented as Hazard ratios (HRs) and 95% confidence intervals (CIs). SPSS 20.0 was used for statistical analysis. *P*-values < 0.05 were reckoned to be statistically significant.

## Results

### Protein expression signature based on IHC distinguished subgroups in PB-DLBCL

**Fig 1:**
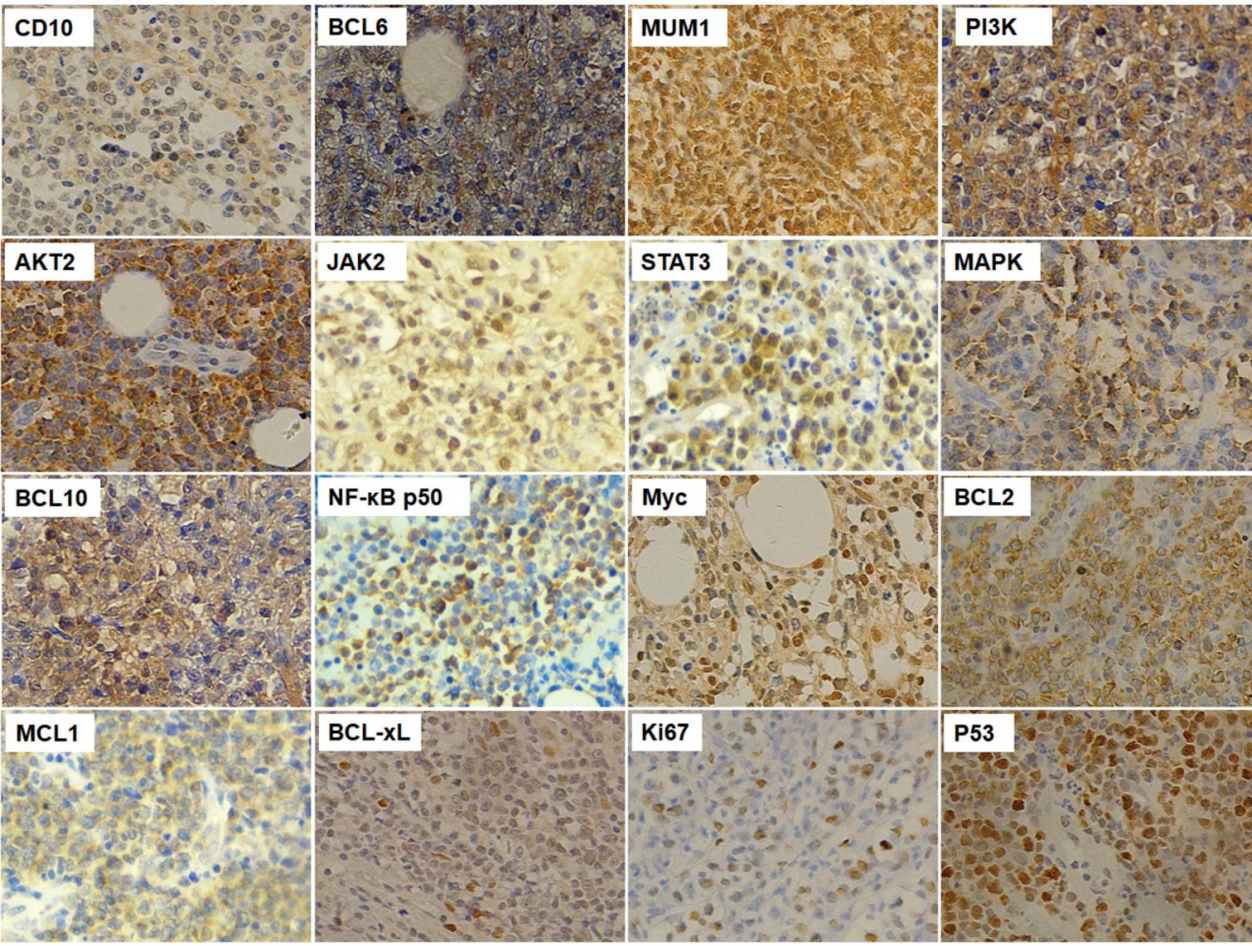
The immunohistochemistry results of CD10, BCL6, MUM1, PI3K, AKT2, JAK2, STAT3, MAPK, BCL10, NF-κB, Myc, BCL2, MCL1, BCL-xL, Ki67 and P53 in PB-DLBCL patients (200×magnification).

In order to comprehensively understand the distinct pathological and molecular characteristics of PB-DLBCL, we firstly screened several general clinical and biological markers of lymphoma cells, and then detected the protein expressions of these markers. First of all, we detected the expressions of CD10 (positive rate: 60.3%), BCL6 (45.6%) and MUM1 (85.3%) to demonstrate the COO classification PB-DLBCL patients according to the Hans method 26. BCR signaling pathway was associated with survival and development of DLBCL cells, and we determined several BCR signaling components, including PI3K (47.1%), AKT2 (41.2%), JAK2 (42.7%), STAT3 (50.0%), MAPK (48.5%), BCL10 (60.3%), and NF-κB p50 (58.8%). Myc together with BCL2 led to a rapidly clinical progression and short survival 33, and the positive rates of expressions of Myc and BCL2 were 66.2% and 67.7% respectively. In addition, we detected the expressions of apoptotic factors, such as MCL1 (22.1%) and BCL-xL (35.3%). the positive expressions of IHC results were shown in Fig. 1.

**Fig 2:**
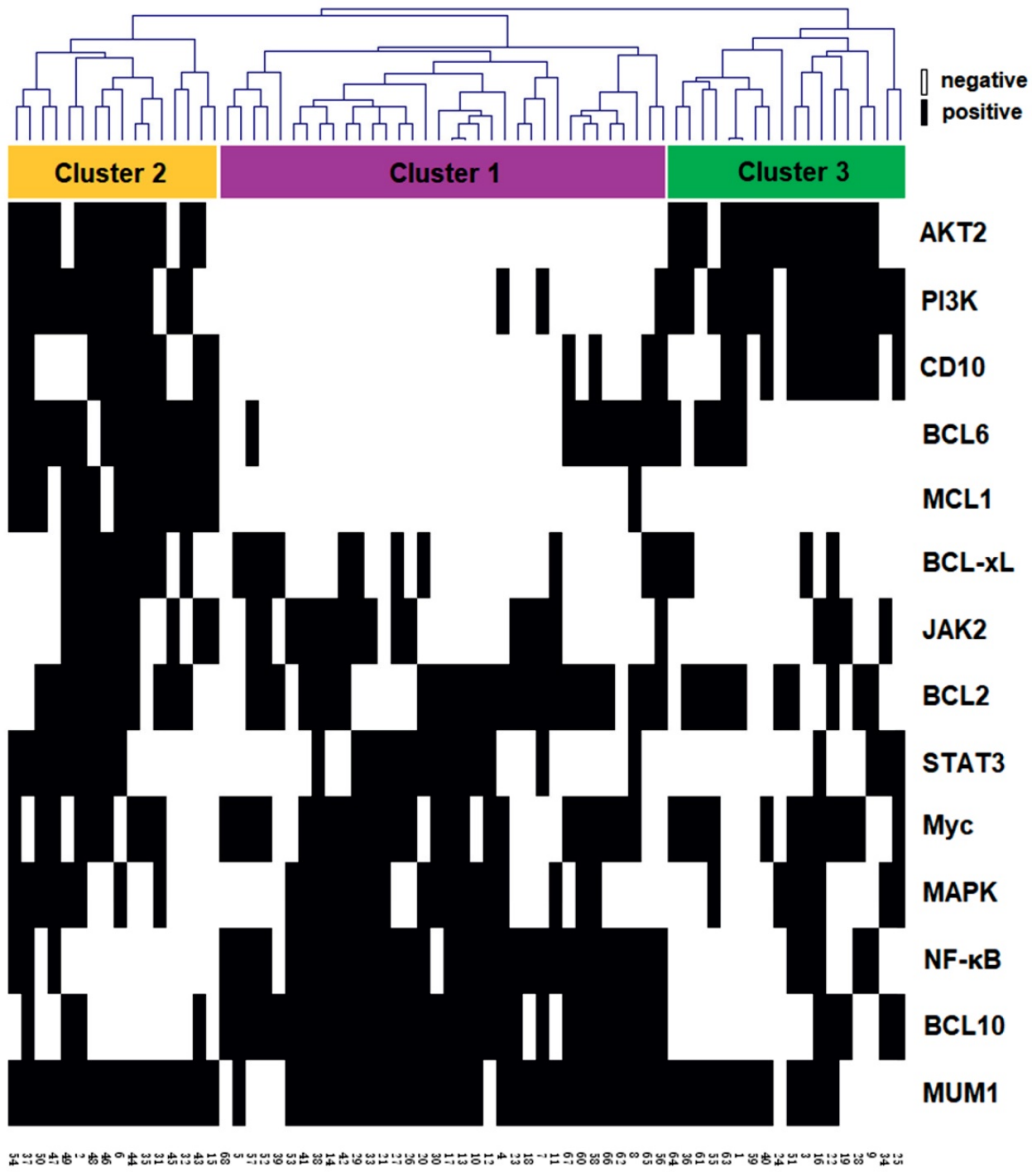
Identification of clusters of PB-DLBCL based on protein expression signatures: hierarchical clustering analysis was performed using all IHC results in the 68 PB-DLBCL samples (columns). Clusters 1-3 with their associated protein expressions were visualized (boxed for each cluster: Cluster 1, purple; Cluster 2, yellow; Cluster 3, green). The positive expression of each protein was presented as black, whereas negative expression was white.

It has been reported that the diversity and complexity of BCR signaling pathway contributed to the biological heterogeneity of DLBCL. To examine whether significant expression patterns of BCR signaling components were informative that they were able to distinguish characteristic subtypes of PB-DLBCL, the protein expression data was hierarchically clustered, as shown in Fig. 2. The vertical columns represented the associated biomarkers whereas the horizontal rows represented 68 samples. According to our clustering result, we identified three robust subsets that differentially expressed protein profiling in PB-DLBCL patients, named Cluster 1, 2 and 3 (Fig. 2).

Cluster 1. In a total of 68 cases, half of the PB-DLBCL patients were included into this cluster, with high NF-κB expression predominantly. These samples also had increasing expression of BCL10, which was an important component of the CBM adapter complex combined with CARD11 and MALT1 that recruited and activated IκB kinase, a key activator of the NF-κB signaling pathway 34. Therefore, we defined this cluster as NF-κB-dependent cluster. Other candidate proteins including Myc and BCL2 also were also significantly higher in this cluster, and tumors with co-occurring Myc and BCL2 were significantly more frequent, which might be associated with poor survival in DLBCL (called double-expression lymphoma) 7, 35, 36. In addition, sporadic cases in this cluster had increasing expressions of STAT3 and MAPK in Cluster 1, which might appear as the upstream signaling of the NF-κB.

Cluster 3. The 18 cases of PB-DLBCLs were classified into this cluster, which exhibited significantly strong expressions of PI3K and AKT2 predominantly. Therefore, this cluster was defined as PI3K-dependent cluster. These samples also had increasing expression of CD10, which was the most major determinant of GCB-type of DLBCLs according to the Hans method 26. We thus hypothesized this cluster be in GCB subtype predominantly, probably associated with more favorable survival.

Cluster 2. The remaining 16 PB-DLBCLs were grouped into this cluster, characterized by low or absent NF-κB expressions, but increasing expressions of MCL1 and BCL-xL, indicating a possible anti-apoptotic effect of this cluster. The cases also had enriched expressions of PI3K and AKT2, which might be associated with the anti-apoptotic protein expressions 37. Additionally, several NF-κB-independent BCR-associated signaling pathway members, such as JAK2, STAT3 and MAPK were also enriched in this cluster, Therefore, we considered this cluster as NF-κB-independent cluster with high anti-apoptotic potential plus PI3K signaling, suggesting several specific targeted therapies for this cluster.

### The relationship between protein expressions of the three clusters of the PB-DLBCL patients and the gene-expression profile from TCGA DLBCL database

**Fig 3:**
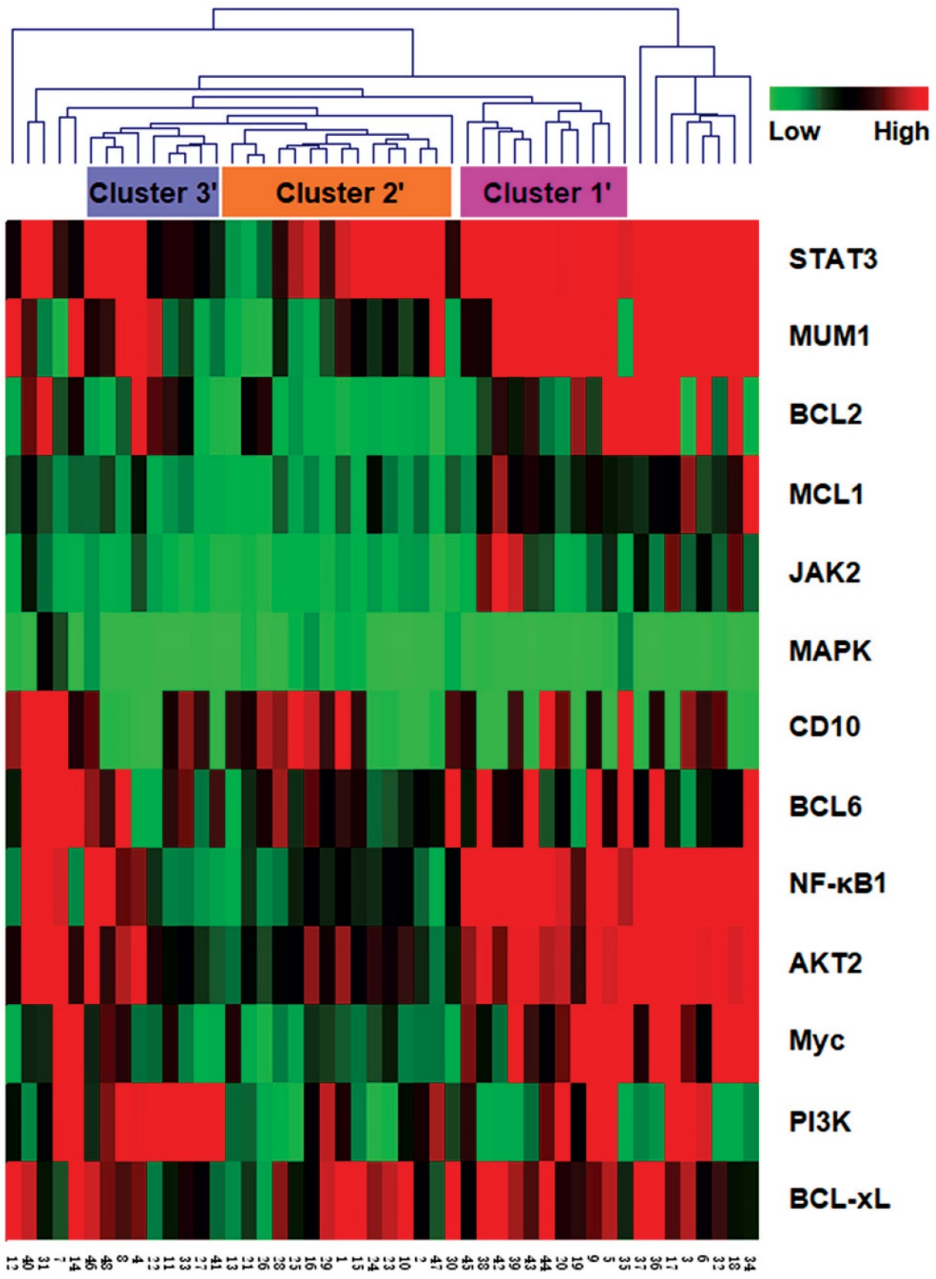
The hierarchical clustering analysis was also carried out to classify the gene expression data of 48 DLBCL patients from TCGA. Cluster 1'-3' were labeled according to the clustering results.

To validate our cluster classification based on the BCR signaling pathway, we collected the DLBCL datasets from the TCGA databases, and examined the associated differential expression data of the BCR signaling pathway members from 48 DLBCL patients accordingly (Fig. 3). The results also demonstrated three clusters (termed Cluster 1', 2' and 3') similar to the above identified clusters, which covered more than 70% of the DLBCL patients. Other cases that were not classified into the clusters might be due to the differences between protein expression and mRNA expression levels.

### Identification of the clusters was associated with different COO subtypes in PB-DLBCL

**Fig 4:**
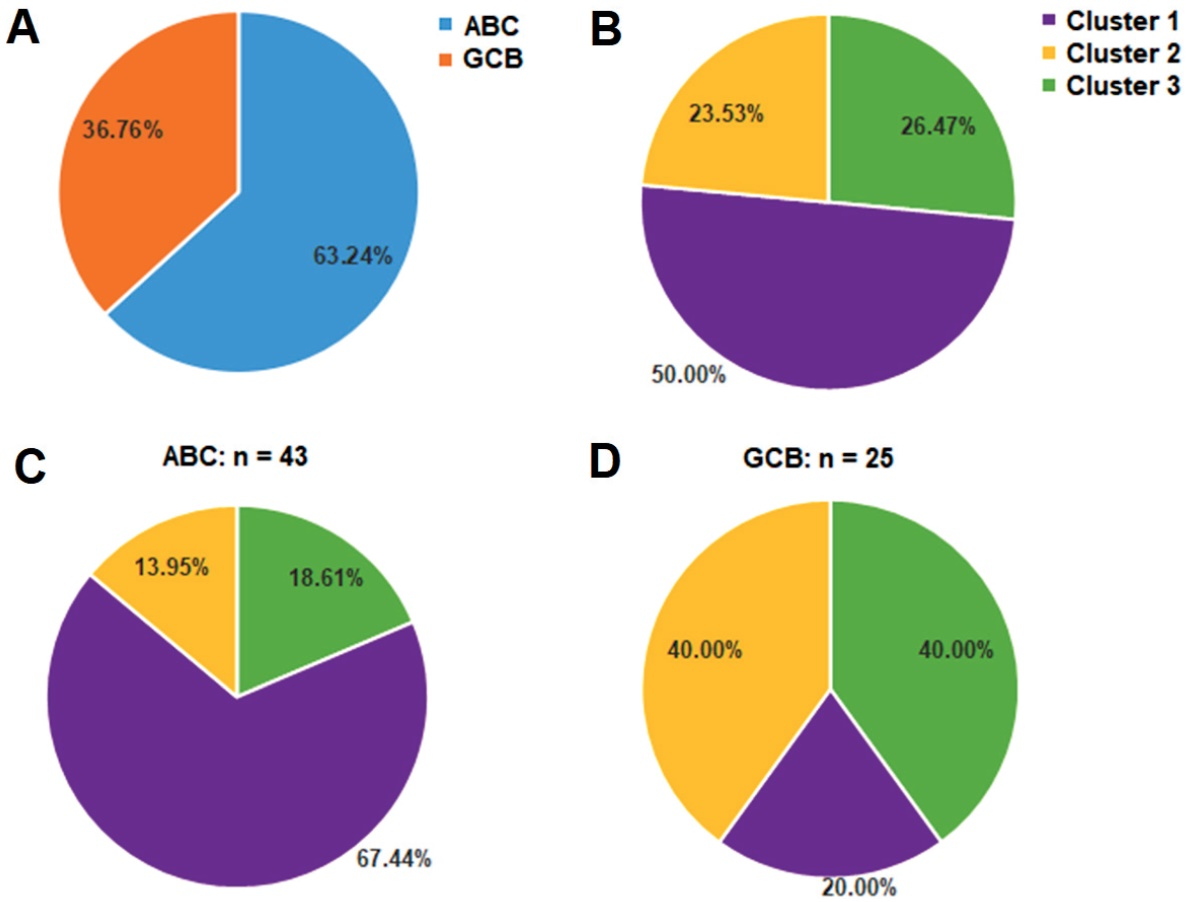
Classification of the three clusters corresponds to different ABC or GCB subtypes of PB-DLBCL: (**A**) the distribution of ABC and GCB subgroups within 68 PB-DLBCL patients; (**B, C, D**) the distribution of Cluster 1-3 within 68 PB-DLBCL patients (**B**), 43 ABC subtype of PB-DLBCL patients (**C**), and 25 GCB subtype of PB-DLBCL patients (**D**).

According to Hans method, CD10, BCL6 and MUM1 expression by IHC analysis has defined two major DLBCL subtypes as ABC and GCB based on the COO classification as mentioned above 26. The majority of PB-DLBCL patients (43 of 68 cases, 63.24%) were classified into ABC subgroup, and 25 cases (36.76%) were GCB (Fig. 4A). To demonstrate whether our identification of the three clusters based on the BCR signaling components (Fig. 4B) was associated with ABC or GCB subgroups, we compared the percentages of the three clusters in the ABC and GCB subgroups respectively. The Cluster 1 was dominated by ABC cases, accounting for 67.44% of the ABC PB-DLBCL patients (Fig. 4C). However, Cluster 3 included mostly GCB cases (10 of 18 cases, 55.56%), although the percentage of Cluster 3 to GCB cases was 40.00% (Fig. 4D). Additionally, the Cluster 2 was composed by both the ABC and GCB gene expression subgroups (Fig. 4C and 4D). These data demonstrated that our classification based on BCR signaling components might be independent from the traditional COO classification.

### There existed close relationship between the three clusters and baseline clinical features

Baseline characteristics were analyzed in a whole cohort of 68 patients diagnosed with PB-DLBCL, as shown in Table 1. All patients were females, and the median age at diagnosis was 50 years (range from 28-80 years). The right breast was involved in 29 patients (42.65%), and the B symptoms were absent in 64 patients (94.12%). The Eastern Cooperative Oncology Group (ECOG) performance status was 0 or 1 in 62 patients (91.18%). Most of the patients (67.65%) had Ann Arbor stage IE. Serum lactate dehydrogenase (LDH) was elevated in 14 (20.59%) of 68 patients. Regarding as the IPI score, the majority of the patients (92.65%) were in the low risk group. Ki67 expressions were elevated in most of the patients (76.47%), and P53 were overexpressed in over half of the patients (55.88%). We also analyzed the correlation between our identified three clusters and the clinicopathologic features. As shown in Table 1, the clusters were significantly associated with the LDH level (*P* = 0.019) and the Ki67 expressions (*P* = 0.017).

### Clinical outcomes of the PB-DLBCL patients in different clusters

**Fig 5:**
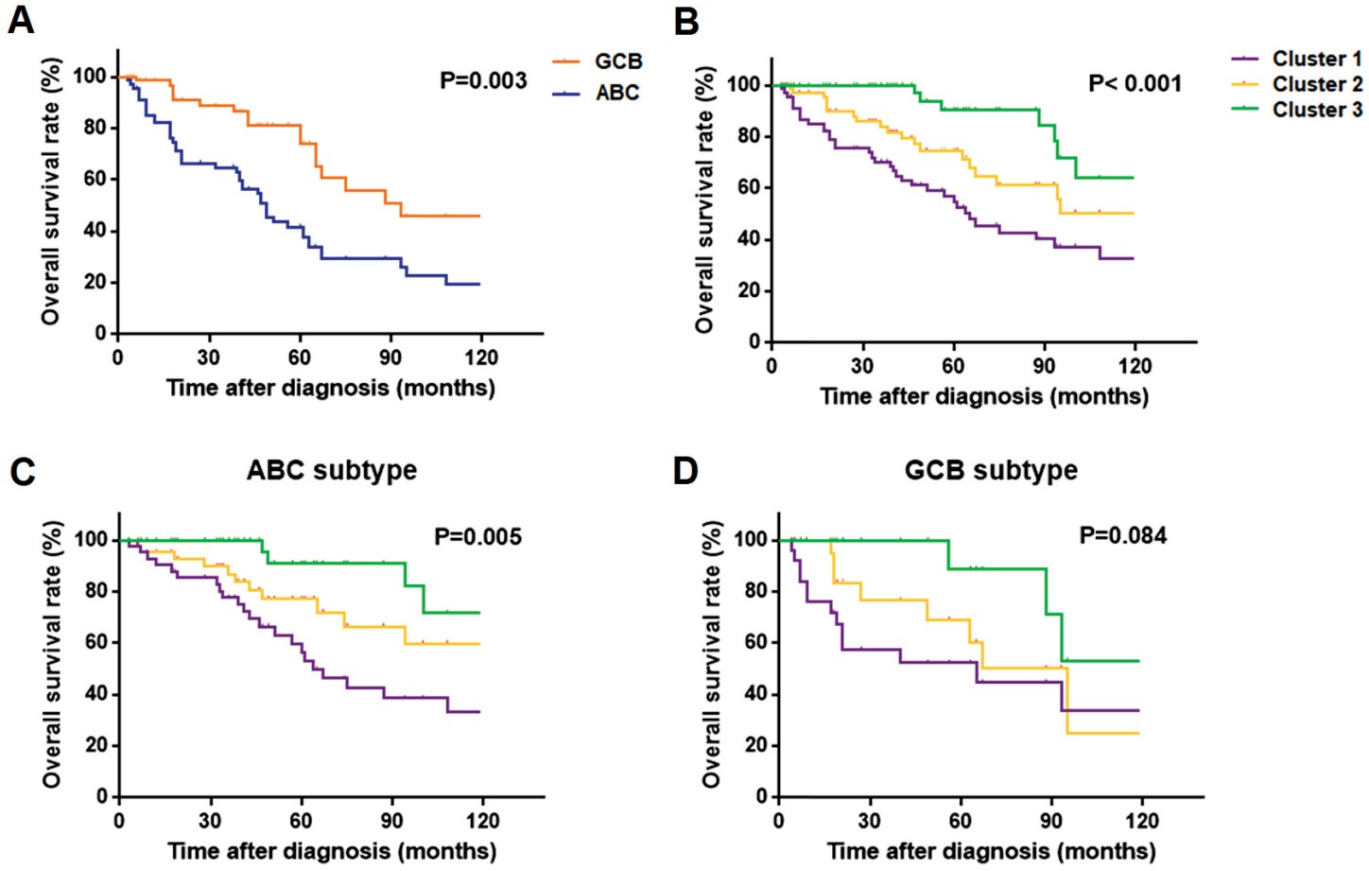
Kaplan-Meier models of overall survival according to ABC or GCB subgroups as well as the Cluster 1-3: (**A**) overall survival rates by ABC and GCB subgroups of 68 PB-DLBCL patients; (**B, C, D**) overall survival rates by Cluster 1-3 of 68 PB-DLBCL patients (**B**), 43 ABC subtype of PB-DLBCL patients (**C**), and 25 GCB subtype of PB-DLBCL patients (**D**).

For the survival analysis, we selected all 68 PB-DLBCL patients with outcome data. Fig. 5A showed a better OS rate of GCB subgroup compared with the ABC group (*P* = 0.003), as previously reported. The three subtypes based on our clustering analysis differed significantly in OS, with the Cluster 3 patients having much more favorable outcomes than the other two groups (*P* < 0.001), and Cluster 1 patients having the worst survival outcome (Fig. 5B). Within ABC PB-DLBCL patients in Fig. 5C, there existed the similar significant OS rates of the three subgroups, with *P*-value as 0.005. Regarding as only 25 PB-DLBCL patients in GCB subgroup, although no significant result was obtained, there was a trend toward favorable OS among patients with Cluster 3 as compared with patients with Cluster 1 and 2 subtypes (Fig. 5D). The distinct prognosis regarding our protein expression subsets of BCR components provided the basis of assessing the prognosis of the PB-DLBCL patients. In addition, the identification of the three clusters was considered as an independent prognostic marker for OS (*P* = 0.013, HR = 0.413; 95%CI: 0.206-0.830), as shown in Table 2. The expression of P53 could also be a prognostic factor in PB-DLBCL patients (Table 2).

**Fig 6:**
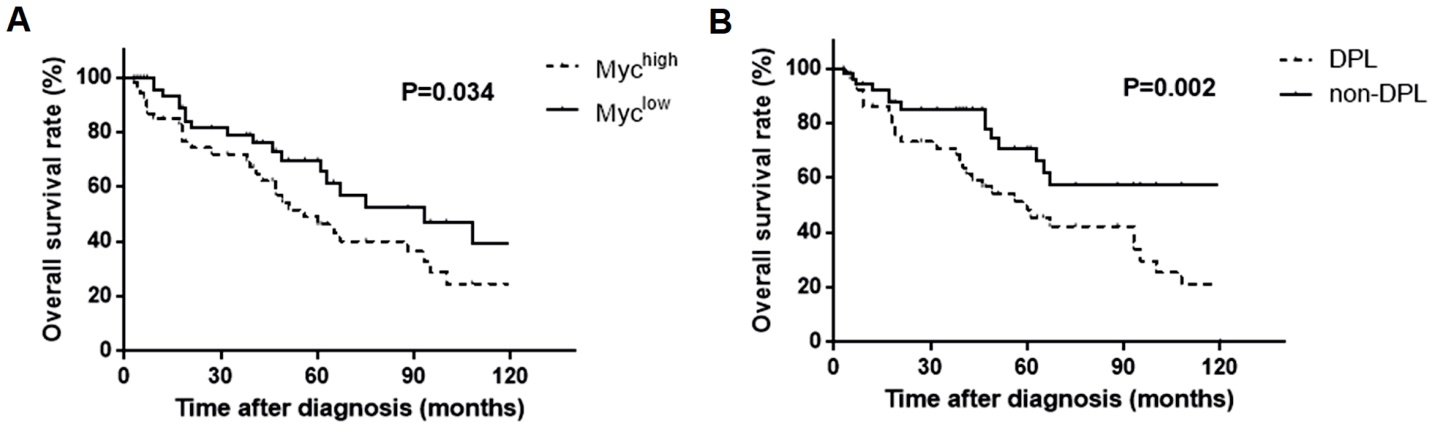
Kaplan-Meier models of overall survival according to Myc expression (**A**) and the double expressions of Myc and BCL2 (DPL) (**B**).

It was noteworthy that Myc expression (66.18%) in our study was significantly higher in PB-DLBCL patients than DLBCL patients (30% of MYC gene expression in TCGA database; 20-30% of Myc protein expression as previously reported). Overexpression of Myc usually resulted in poor prognosis of lymphoma, and this effect can be augmented among tumors that co-expressed BCL2 (DPL) 7, 35, 36. Therefore, we further performed the survival analysis, and the results showed that patients with higher Myc expression and double expressions of Myc and BCL2 were associated with a significantly worse survival rates (*P* = 0.034, Fig. 6A; *P* = 0.002, Fig. 6B respectively), suggesting Myc together with BCL2 probably played a role in PB-DLBCL besides BCR signaling components.

## Discussion

The robust protein expression signature for PB-DLBCL that we presented in this study showed a novel landscape of the biological heterogeneity of the PB-DLBCL determined by BCR signaling pathway and the characteristic attributes of our identified three clusters of PB-DLBCLs that might influence the clinical outcomes. Our studies provided the basis of understanding the clinical complexity of PB-DLBCLs, which could also contribute to the development of specific therapeutic targets for PB-DLBCL patients in the future.

Despite that COO classification of ABC and GCB subtypes has been widely used to distinguish DLBCL cells to predict survival outcomes of patients, it may not comprehensively demonstrate the distinct biological feathers of all DLBCLs, especially in several special types of lymphomas 8, 21. PB-DLBCL is a rare subtype of DLBCL derived from extranodal tissues with poor survival 10, 11, 13. Although a series of evidences showed its predominance of ABC subtype, the PB-DLBCL patients also exhibited clinical heterogeneity in therapeutic responses and survival outcomes 13, 16. Furthermore, limited studies have focused on PB-DLBCL's biological diversity, optimal therapeutic regimens, as well as prognosis 10-12, 17. Therefore, novel classification approaches are required to reclassify PB-DLBCLs to better understand this specific type of extranodal lymphoma.

BCR signaling pathway is central to the pathogenesis and development of B cell malignancies, and is composed by many pathways and transcriptional networks 18. Generally, the BCR signaling can be divided into the upstream and downstream signaling pathways. The upstream BCR signaling engages the activation signals, whereas the downstream signaling mediates the functional survival signals, which is more important for research and intervention 20. Furthermore, the complexity and heterogeneity of the BCR signaling pathway components made them possible to distinguish DLBCL cells. In this study, we identified three robust clusters according to BCR signaling components of PB-DLBCL, which were proven to be different from the traditional ABC and GCB catalogue. Notably, our results showed that the ABC and GCB subgroups were both constituted by the three clusters of the PB-DLBCL patients, suggesting a more comprehensive classification intergrating the COO subtypes as well as our clustering analysis based on the BCR signaling pathway members. Moreover, our identified clusters were roughly consistent with the results from gene expression data of TCGA exon, indicating that our results might be implemented clinically.

In our study, the Cluster 1, a genotype that has been characterized by frequent NF-κB expression and activation of the NF-κB-dependent signaling, accounted for nearly a half of the PB-DLBCL patients. In general, NF-κB activation has been considered as an important feature of ABC subgroup of DLBCL, which relies on constitutive NF-κB signaling to reduce apoptosis and sustain viability, called “chronic active” BCR signaling 19, 22. Constitutive activation of NF-κB mediates several downstream survival signals, and inhibition of NF-κB activity by blocking BCR signaling causes cell cycle arrest and apoptosis 38. In our results, the Cluster 1 associated with NF-κB activation was dominated by ABC subtype, which was coincidence with the previous studies related to primary extranodal lymphomas. Furthermore, survival analysis in our study showed that patients in Cluster 1 suffered from the worst survival outcome, probably due to their constitutive activation of the NF-κB pathway, which is considered to weaken the effects of cytotoxic agents. These results might account for the blunt therapeutic response of the majority of PB-DLBCL patients in clinic, demonstrating that NF-κB-dependent signaling pathway might be an important intervention target. In addition, the Cluster 1 included cases with both Myc and BCL2 overexpression also provided another explanation of poor survival of patients in this group, due to their important role in malignant proliferation of the lymphoma cells 35.

From our clustering results, Cluster 3 was enriched for PI3K/AKT signaling, which also played an important role in mediating survival signals of BCR signaling pathways. In comparison to “chronic active” BCR signaling characterized by NF-κB, the PI3K was considered as another signaling pathway member in “tonic” BCR signaling, where a constitutively active form of PI3K rescued the B-cell survival while surface BCR expression was genetically ablated 18. Although the PI3K/AKT also played a role in chronic BCR signaling ABC subtype to indirectly modulate downstream NF-κB signals, most PI3K activation by tonic BCR signaling occurs in GCB tumors, which usually had an improved clinical outcome compared with ABC DLBCLs 38, 39. As expected, PB-DLBCL patients in Cluster 3 included mostly GCB cases (70%), with the much more favorable outcomes than the other two groups, demonstrating that this cluster driven by PI3K-dependent signaling might have a response to therapy.

Besides the above two clusters with specific characters, we also defined a group of PB-DLBCL patients with shared molecular features using our clustering map, as Cluster 2. The Cluster 2 subtype showed unique features compared with Cluster 1 and 3, and more importantly, exhibited a new and evolving definition of a cluster group of PB-DLBCLs. Firstly, this group lacked the single ABC and GCB characters, but combined some features of both subtypes from our results. Secondly, this cluster shed light on non-NF-κB-mediated BCR signaling pathways, such as JAK/STAT, MAPK, but enriched anti-apoptotic factors MCL1 and BCL-xL, suggesting that there might be other mechanisms regulating the survival signals in this cluster. Last but not least, this cluster combined PI3K/AKT signaling pathways, made this cluster showed some similarities from Cluster 3, and this results might explained the up-regulation of the anti-apoptotic expressions as previously reported 37. Taken together, this cluster could be characterized by an NF-κB-independent anti-apoptotic subgroup plus PI3K signaling, which displayed distinctive features from both Cluster 1 and 3 classifications in biological behavior. In addition, the survival analysis in our studies showed that PB-DLBCL patients in Cluster 2 had medium survival rates between Cluster 1 and 3, which was consistent with its biological characteristics.

## Conclusions

In conclusion, our clustering studies to identify three robust PB-DLBCL clusters distinctively according to BCR signaling components presented a novel signature for assessing previously unrecognized protein expression subsets. These findings in our study provided two important implications in PB-DLBCL patients. First, the distinct protein expression subtypes driven by different BCR signaling pathways might contribute to the subsequent biological behavior of the lymphoma cells. Second, each cluster had distinct survival outcomes after therapy and probably guided to the selection of targeted therapies owing to their distinct signaling abnormalities.

## Supplementary Material

Supplementary figure and table.

## Figures and Tables

**Table 1:** Clinicopathologic features of PB-DLBCL patients according to the three clusters

Characteristics	n=68 (%)	Cluster 1 (n=34)	Cluster 2 (n=16)	Cluster 3 (n=18)	*P*-value
Age					
< 50 years	45 (66.18)	21	14	10	0.108
≥ 50 years	23 (33.82)	13	2	8	
Laterality					
Right	29 (42.65)	13	10	6	0.175
Left	39 (57.35)	21	6	12	
B symptoms					
Absent	64 (94.12)	32	15	17	0.996
Present	4 (5.88)	2	1	1	
ECOG PS					
0	44 (64.71)	20	10	14	0.410
1	18 (26.47)	12	4	2	
≥ 2	6 (8.82)	2	2	2	
Stage					
I_E_	46 (67.65)	24	11	11	0.781
II_E_	22 (32.35)	10	5	7	
LDH					
Normal	54 (79.41)	28	9	17	0.019
Elevated	14 (20.59)	6	7	1	
Adjusted IPI					
0	35 (51.47)	16	8	11	0.800
1	28 (41.18)	14	7	7	
2	4 (5.88)	3	1	0	
3	1 (1.47)	1	0	0	
Ki67 expression					
< 70%	16 (23.53)	5	8	3	0.017
≥ 70%	52 (76.47)	29	8	15	
P53 expression					
<30%	30 (44.12)	14	8	8	0.842
≥ 30%	38 (55.88)	20	8	10	

*ECOG PS* Eastern Cooperative Oncology Group performance status, *LDH* lactate dehydrogenase,* IPI* international prognostic index

**Table 2:** Multivariate analysis of prognostic factors for survival in PB-DLBCL patients

Covariate	OS		
	HR	95%CI	*P*-value^a^
Age, y	1.022	0.391-2.672	0.965
Laterality	0.830	0.392-1.759	0.627
B symptoms	0.532	0.064-4.388	0.558
ECOG PS	0.838	0.435-1.617	0.599
Stage	2.254	0.797-6.376	0.126
LDH	0.649	0.156-2.709	0.553
Adjusted IPI	1.214	0.454-3.242	0.699
Ki67	2.021	0.554-7.376	0.287
P53	3.599	1.465-8.839	0.005
Myc	2.742	0.648-11.611	0.171
DPL	0.606	0.228-1.611	0.315
Three clusters	0.413	0.206-0.830	0.013

^a^Cox analysis

## Abbreviations

PB-DLBCL: Primary breast diffuse large B-cell lymphoma
BCR: B-cell receptor
COO: cell-of-origin
ABC: activated B-cell like
GCB: germinal center B-cell like
IPI: International Prognostic Index
OS: overall survival
CARD11: caspase recruitment domain family member 11
PTK: protein tyrosine kinases
WHO: World Health Organization
IHC: Immunohistochemistry
MEV: Multi Experiment View
ECOG: Eastern Cooperative Oncology Group
LDH: lactate dehydrogenase
