# Supplementary material for: A novel classification based on B-cell receptor signal gene expression correlates with prognosis in primary breast diffuse large B-cell lymphoma
Source: J Cancer. 2020 Feb 10;11(9):2431–41. doi: 10.7150/jca.39083 (PMC7066002; doi:10.7150/jca.39083)
Supplement: Supplementary file 1 — Supplementary figure and table. [file jcav11p2431s1.pdf]

Supplement Figure

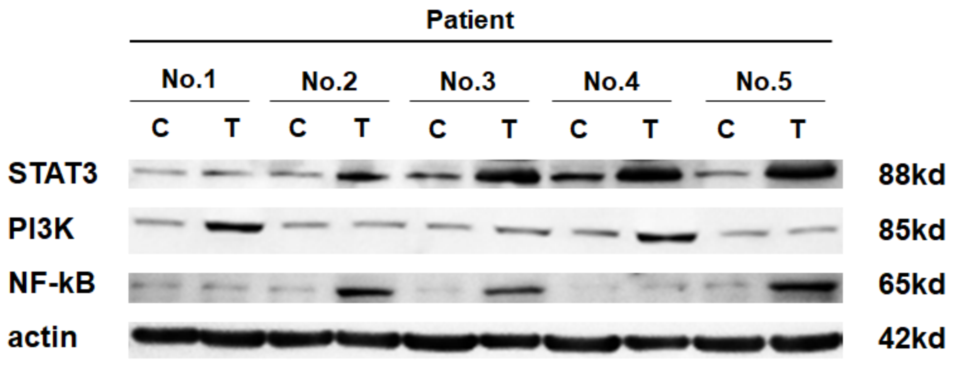

Table S1. Validation IHC results with WB method

Sample	PI3K		STAT3		NF-κB	
	IHC	WB	IHC	WB	IHC	WB
1	+	+	-	-	-	-
2	-	-	+	+	+	+
3	-	-	+	+	+	+
4	+	+	+	+	-	-
5	-	-	+	+	+	+
